# Supplementary material for: A qualitative exploration of young people’s experiences of attempted suicide in the context of alcohol and substance use
Source: PLoS One. 2021 Aug 31;16(8):e0256915. doi: 10.1371/journal.pone.0256915 (PMC8407575; doi:10.1371/journal.pone.0256915)
Supplement: S1 Appendix — (DOCX) [file pone.0256915.s001.docx]

**S1 Appendix. Consolidated criteria for reporting qualitative studies (COREQ): 32-item checklist**

Domain 1: Research team and reflexivity

Personal Characteristics

1. Interviewer/facilitator Which author/s conducted the interview or focus group?
Rebecca Guest (RG) **page 6**

2. Credentials What were the researcher’s credentials? E.g. PhD, MD
DClinPsy **page 7**

3. Occupation What was their occupation at the time of the study?
Trainee Clinical Psychologist **page 7**

4. Gender Was the researcher male or female?
Female **page 7**

5. Experience and training What experience or training did the researcher have?
DClinPsy **page 7**

Relationship with participants

6. Relationship established Was a relationship established prior to study commencement?
No- **page 5**

7. Participant knowledge of the interviewer What did the participants know about the researcher? e.g. personal goals, reasons for doing the research
Participants were made aware, through the participant information sheet **page 5**

8. Interviewer characteristics What characteristics were reported about the interviewer/facilitator? e.g. Bias, assumptions, reasons and interests in the research topic
**Page 7**

Domain 2: study design

Theoretical framework

9. Methodological orientation and Theory What methodological orientation was stated to underpin the study? e.g. grounded theory,discourse analysis, ethnography, phenomenology, content analysis

Phenomenology **page 6**

Participant selection

10. Sampling How were participants selected? e.g. purposive, convenience, consecutive, snowball
Purposive **page 5**

11. Method of approach How were participants approached? e.g. face-to-face, telephone, mail, email
They were approached via their care team/ care coordinator and asked whether they would like to take part face to face or via telephone **page 5**

12. Sample size How many participants were in the study?
7 **Page 7**

13. Non-participation How many people refused to participate or dropped out? Reasons?
1 participant agreed to take part but at initial screening she had attempted to take her life too recently to take part in the study. The interview was terminated and this was also communicated with her care team. **N/A**

Setting

14. Setting of data collection Where was the data collected? e.g. home, clinic, workplace

**Page 6**

15. Presence of non-participants Was anyone else present besides the participants and researchers?
In one interview the participants partner attended for moral support. He did not take part in the interview.

**Page 8**

16. Description of sample What are the important characteristics of the sample? e.g. demographic data, date

**Page 8**

Data collection

17. Interview guide Were questions, prompts, guides provided by the authors? Was it pilot tested?
n/a

18. Repeat interviews Were repeat interviews carried out? If yes, how many?
No

19. Audio/visual recording Did the research use audio or visual recording to collect the data?
Audio **Page 6**

20. Field notes Were field notes made during and/or after the interview or focus group?
observations and reflections notes were taken during and after the interviews **Page 7**

21. Duration What was the duration of the interviews or focus group?
40-75 minutes

22. Data saturation Was data saturation discussed?
n/a

23. Transcripts returned Were transcripts returned to participants for comment and/or correction?

No- due to time constraints

Domain 3: analysis and findings

Data analysis

24. Number of data coders How many data coders coded the data?

1 **page 7**

25. Description of the coding tree Did authors provide a description of the coding tree?
n/a

26. Derivation of themes Were themes identified in advance or derived from the data?
derived from the data **Page 7**

27. Software What software, if applicable, was used to manage the data?
n/a

28. Participant checking Did participants provide feedback on the findings?
no- due to time constraints, however they were sent a copy of the summarised findings

Reporting

29. Quotations presented Were participant quotations presented to illustrate the themes / findings? Was each quotation identified? e.g. participant number

Yes **Pages 11-23**

30. Data and findings consistent Was there consistency between the data presented and the findings?
yes  **Pages 11-23**

31. Clarity of major themes Were major themes clearly presented in the findings?
yes **Pages 11-23**

32. Clarity of minor themes Is there a description of diverse cases or discussion of minor themes?
yes **Pages 11-23**
