# Supplementary figures and images for: A qualitative exploration of young people’s experiences of attempted suicide in the context of alcohol and substance use
Source: PLoS One. 2021 Aug 31;16(8):e0256915. doi: 10.1371/journal.pone.0256915 (PMC8407575; doi:10.1371/journal.pone.0256915)

**S2 Appendix. Recruiter information sheet**


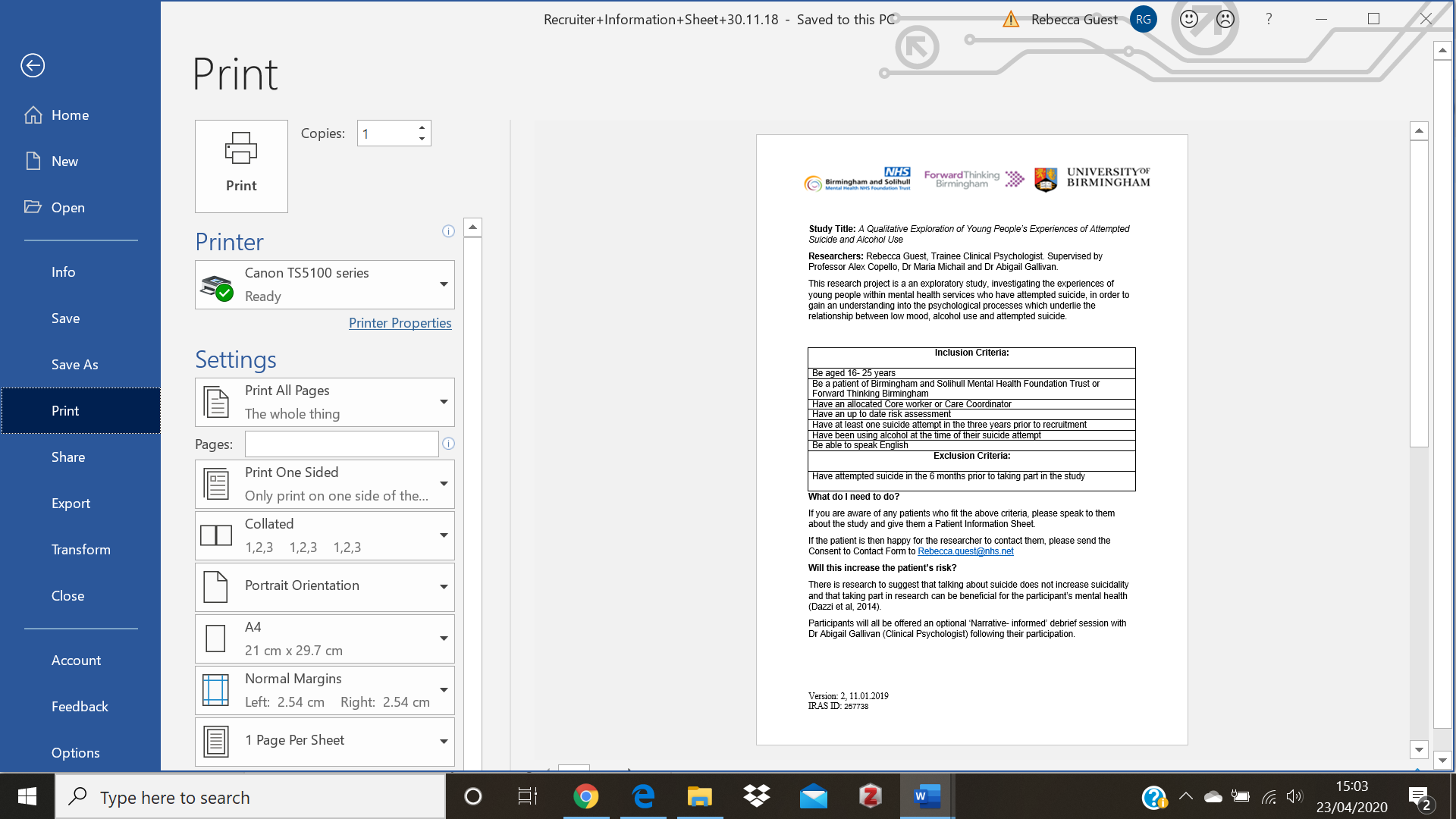

Supplement: S2 Appendix — (DOCX) [file pone.0256915.s002.docx]

**S4 Appendix. Consent for researcher contact form**


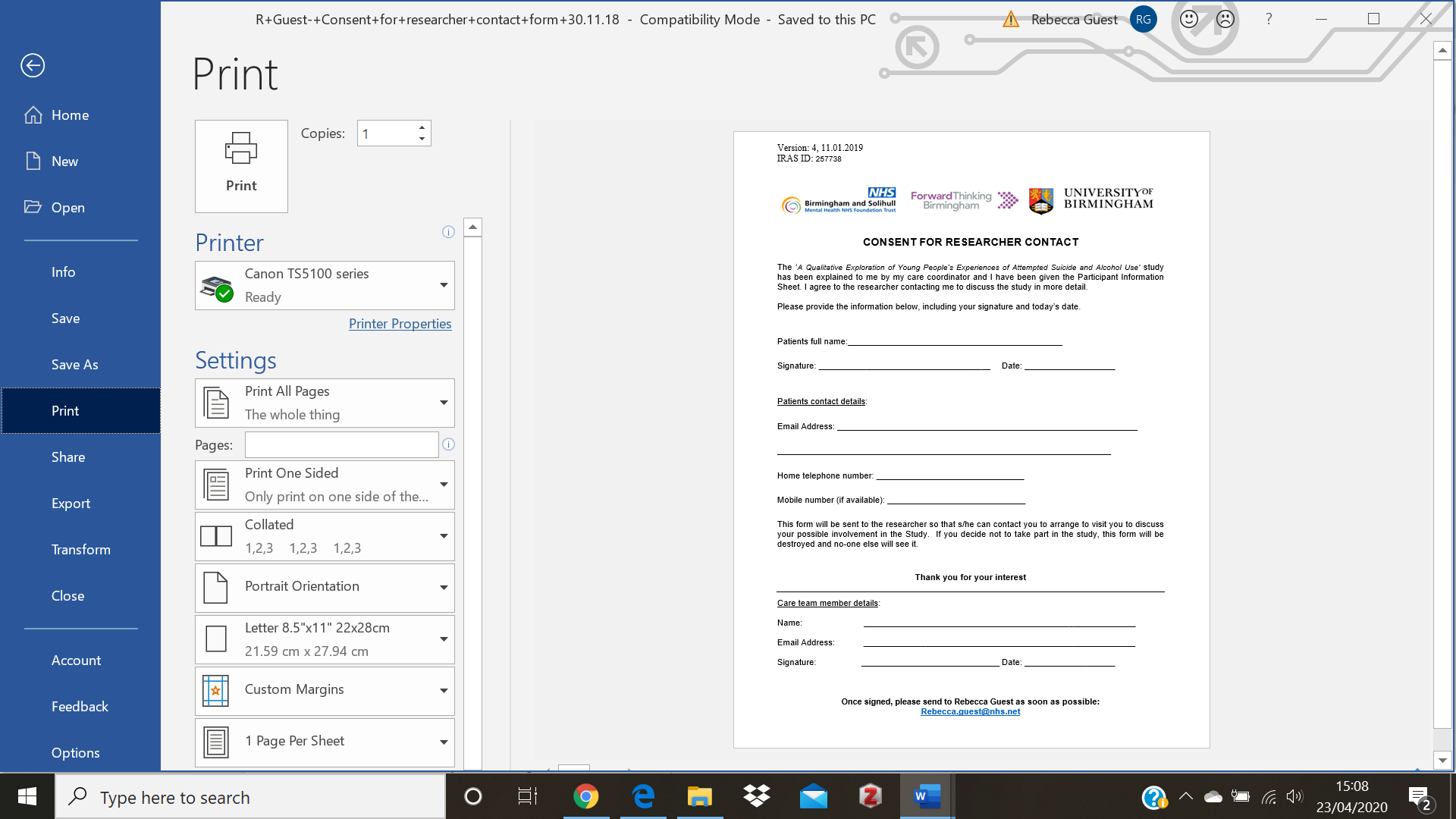

Supplement: S4 Appendix — (DOCX) [file pone.0256915.s004.docx]
