# Supplementary material for: A qualitative exploration of young people’s experiences of attempted suicide in the context of alcohol and substance use
Source: PLoS One. 2021 Aug 31;16(8):e0256915. doi: 10.1371/journal.pone.0256915 (PMC8407575; doi:10.1371/journal.pone.0256915)
Supplement: S3 Appendix — (DOCX) [file pone.0256915.s003.docx]

**S3 Appendix. Recruitment Poster**

**Discussing experiences of alcohol use and**

**attempts to end your life**

**We are looking for participants to take part in some**

**research**



**Aged 16**

**-**

**25**



**History of attempted suicide in the last three years**



**Currently involved with Mental Health services**



**Would say that you have had ‘problematic use ‘ of**

**alcohol**

**This would involve:**



**A meeting to discuss the research (approx. 60 mins)**



**A recorded interview (60**

**-**

**90**

**mins**

**)**



**An**

**optional**

**debrief session with a Clinical Psychologist**


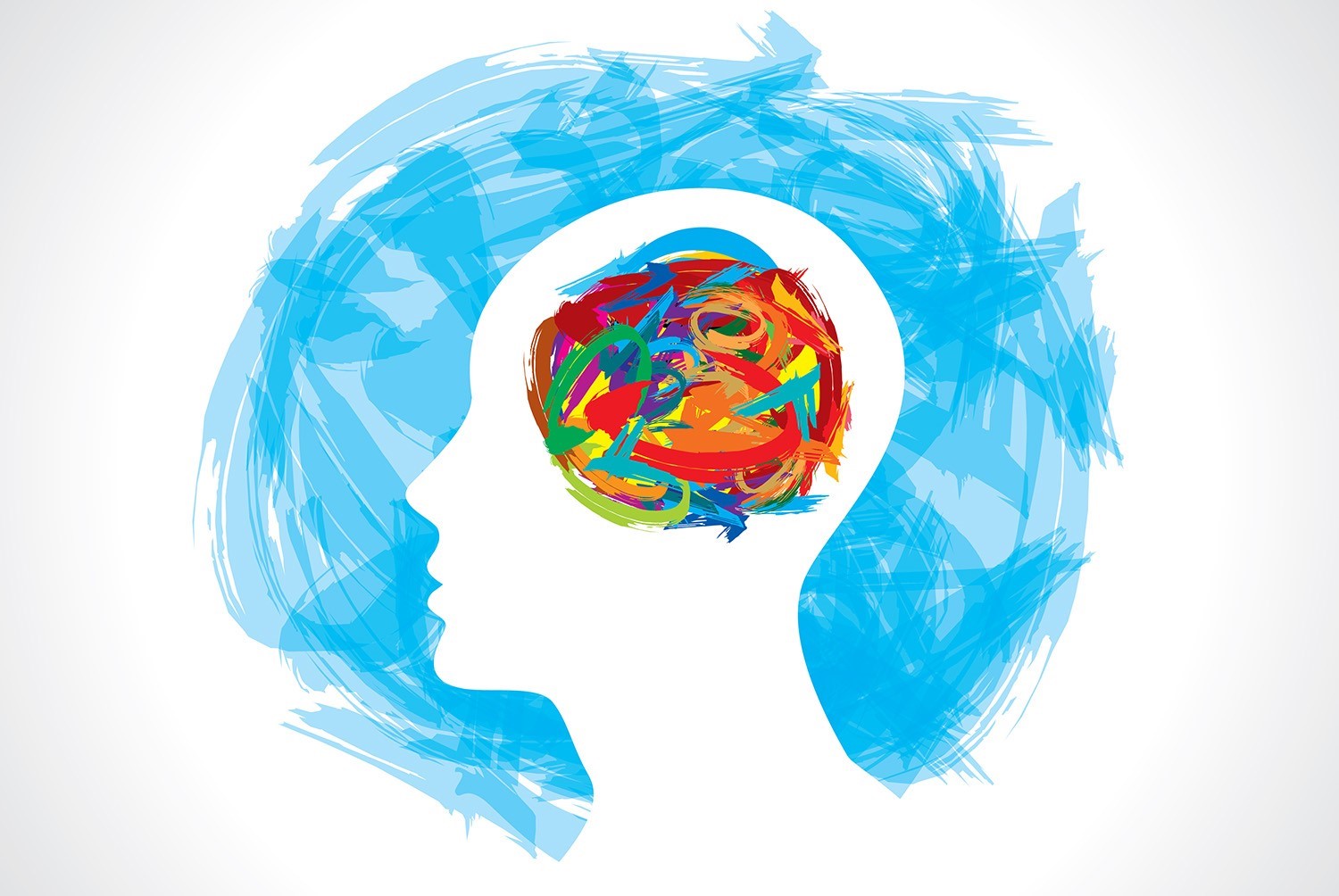


If you would like to find out more:

Rebecca Guest

Trainee Clinical Psychologist

University of Birmingham

**Rebecca.guest@nhs.net**

**Could you help us understand if**

**there are any links between alcohol**

**use and attempted suicide?**

As a thank you for taking part in this study, we

will offer every participant a £10 voucher
