# Supplementary material for: A qualitative exploration of young people’s experiences of attempted suicide in the context of alcohol and substance use
Source: PLoS One. 2021 Aug 31;16(8):e0256915. doi: 10.1371/journal.pone.0256915 (PMC8407575; doi:10.1371/journal.pone.0256915)
Supplement: S5 Appendix — (DOCX) [file pone.0256915.s005.docx]

**S5 Appendix. Participant information sheet**

**Participant Information Sheet**

**Study Title:** *A Qualitative Exploration of Young People’s Experiences of Attempted Suicide and Alcohol Use*

**Researchers:** Rebecca Guest, Trainee Clinical Psychologist. Supervised by Professor Alex Copello, Dr Maria Michail and Dr Abigail Gallivan

You are being asked to take part in a research project being completed as part of a Doctorate in Clinical Psychology at the University of Birmingham. Before you decide whether or not to take part, it is important that you read the following information which will help you to understand why the research is being done and what taking part will involve. Please feel free to ask any questions you may have about the information you read.

**What’s the purpose of the study?**

This research project hopes to interview young people (aged 16-25) who have had personal experiences of attempting suicide alongside the use of substances or alcohol. This is to help us to get an understanding of the psychological processes which might lead up to an individual attempting to take their own life and how the use of alcohol may or may not impact on this.

**What would taking part involve?**

If you agree to take part in the study, you will be given a consent form to sign. Once you have given your consent, you will be asked to complete a questionnaire about which substances you may or may not have use in the past two years.

You will then be asked to arrange a time that is convenient for you to attend an interview of around 60 minutes. In this interview, we are interested to hear about your experience(s) of attempting to take your own life alongside the use of substance or alcohol, the build up to this and your reflections following this.

**What are the possible benefits of taking part?**

The research tells us that talking about our experiences can be beneficial to our mental health. Taking part in this research may give you some space to think reflectively about your experiences. You will be helping to inform our understanding of what can be an exceptionally difficult time for individuals, as well as potentially helping to guide interventions and future risk management procedures. You will be given the opportunity to have an optional one off “Narrative therapy-informed” debrief session with a Clinical Psychologist following your participation. Narrative therapy seeks to be a respectful, non-blaming approach and centres people as the experts in their own lives. It views problems as separate from people and assumes people have many skills, competencies, beliefs, values, commitments and abilities that will assist them to reduce the influence of problems in their lives (Dulwich Centre).

**What are the possible disadvantages or risks of taking part?**

The topic which we will be discussing during the interview can be understandably upsetting for people, if this were to happen during the interview, you will be given a break or the opportunity to terminate the interview. You can also choose to withdraw at any time without giving a reason. You will be given debrief information and the option to attend an individual debrief session with a Clinical Psychologist.

**What will happen if I do not wish to take part?**

If you do not wish to take part in the study, this will not impact on your on-going care in anyway. If you do choose to participate and then wish to withdraw at a later date, you can withdraw all of your data up to two weeks following your participation, without giving a reason.

**What will happen to my data?**

The University of Birmingham is the sponsor for this study based in the United Kingdom. We will be using information from you in order to undertake this study and will act as the data controller for this study. This means that we are responsible for looking after your information and using it properly. The University of Birmingham will securely keep identifiable information about you for 10 years after the completion of the study.

Your rights to access, change or move your information are limited, as we need to manage your information in specific ways in order for the research to be reliable and accurate. If you withdraw from the study, we will keep the information about you that we have already obtained. To safeguard your rights, we will use the minimum personally-identifiable information possible.

The University of Birmingham will keep your name and contact details (email address) confidential and will not pass this information to any other organisation. The University of Birmingham will use this information as needed, to contact you about the research study, and make sure that relevant information about the study is recorded for your care, and to oversee the quality of the study. My Supervisors from The University of Birmingham may look at your research records to check the accuracy of the research study. The University of Birmingham will only receive information without any identifying information. The people who analyse the information will not be able to identify you and will not be able to find out your name, or contact details.

Your consent form will be locked in a secure filing cabinet and kept in accordance with data protection principles, for 10 years. The interview will be recorded on an encrypted Dictaphone, this data will then be transcribed verbatim and anonymised. The audio file will then be deleted.

You can choose to remove your data for up to two weeks following your interview, after this time you data will not be able to be removed due to the data analysis process. However, at your request the researcher can remove any direct quotes from the report.

All personal data and research data will be stored separately and only approved members of the research team will have access to that data. All information and data will be kept confidential. However within the report write up, some direct quotes from your interview may be used, at your request these direct quotes can be removed.

**Confidentiality**

*In line with confidentiality principles everything discussed will be kept confidential, unless the researcher becomes worried about your own safety or the safety of anyone else, at which point they will share this information with your Care Coordinator and the Research Supervisors.*

**What happens to the results?**

The research study is due to be completed by September 2020. It is expected that the results will be published in a peer-reviewed journal of which you will receive a copy if you wish. As it can take time for research papers to get published, we can circulate a report containing the results to you, should you wish. There will be no personally identifiable information published within the report.

**Further information**

Thank you for taking the time to read this information and I hope that you will consider taking part in this research project.

If you have any questions about what you have read, please contact:

Rebecca Guest

Email: [Rebecca.guest@nhs.net](mailto:Rebecca.guest@nhs.net)

Postal Address: School of Psychology,
University of Birmingham,
52 Pritchatts Road, B15 2SA

If you would like to make a complaint about any part of the research process, you can contact with Dr Maria Michail ([m.michail@bham.ac.uk](mailto:m.michail@bham.ac.uk)), Professor Alex Copello ([a.g.copello@bham.ac.uk](mailto:a.g.copello@bham.ac.uk)) or the Research Governance Team at University of Birmingham ([researchgovernance@contacts.bham.ac.uk](mailto:researchgovernance@contacts.bham.ac.uk))
