# Supplementary material for: A qualitative exploration of young people’s experiences of attempted suicide in the context of alcohol and substance use
Source: PLoS One. 2021 Aug 31;16(8):e0256915. doi: 10.1371/journal.pone.0256915 (PMC8407575; doi:10.1371/journal.pone.0256915)
Supplement: S6 Appendix — (DOCX) [file pone.0256915.s006.docx]

| **S6 Appendix. Additional quotes** | | |
| --- | --- | --- |
| **Superordinate themes** | **Subordinate themes** | **Additional quotes** |
| 1. The complexity of relationships | 1.1 Keeping safe from others  1.2 Still needing a connection despite the difficulties | Annie: “you know my sister she was a trigger, my mom, not sure much my dad, but then school life, the people at school, the teachers more than anything, they were the triggers and right now its my brother who’s the trigger”  Annie: “got a boyfriend, got into an abusive relationship, he used to beat me, he said he had an attachment disorder, if I would refuse to go he would tell me to kill myself, slit my throat, slit my wrists. So I would go and then get beat up and now it’s my little brother beating me up and saying nasty shit”  Liz: “like when I was young, my mom and dad used to argue all the time, like in front of me and stuff which wasn’t very nice, and it affected me and my brothers quite a bit, I think it was me more than my brothers because I was the older one, and they didn’t really understand what was going on”  Sarah: “its that and then, my dad and my brother arguing about little things and very kind of noisy household with large personalities [ok] urm, so it its, the kind of family that would be stressful for anyone, but then on top of that you’ve got my mom trying to get me to drink so its very much, I have, like I have to prepare myself to go over and say no.”  Sarah: “well I’ve spoken to her about it before and you now, but she enabled it quite a lot and she was very much in the mind set of, its s fun, likes to get drunk kind of thing, urm but I have spoken to her about it before but it’s just kind of in one ear out the other”  Matt: “but if I wasn’t put into certain scenarios I wouldn’t have got angry [yeah] and it just goes towards my frustrations with the system and how people shouldn’t be put through that cus especially myself, it deteriorated my mental health quite bad cus I’ve always had my kind of frustrations about the mental health system and everything that has happened to me ur, I’ve had quite bad experiences with mental health professionals [right] urr, [in what way?] urm, I think its, at the time, I knew what I needed and everyone like in my family knew what I needed, the, there was a couple of times where doctors would refuse treatment, I was having home treatment coming out to the house and they were just like I guess triggering me, several times”  Matt: “I had changed anyway, like, I mean, it was two very different schools so being at the school that I was for two years had kinda changed me a bit [right], I mean a lot more wary of people so”  Louise: “thing is with the memories, the reason why it triggers me so much is because I was involved in domestic violence with my ex-boyfriend”  Louise: “I hated school, I didn’t really get on with anybody urm, I just kind of was by myself and when I was in a group, I would always try and be like urm, the silly one, who always tries to make everyone laugh, just to try and fit in[ok] but it didn’t work so I would I would get, the voices in my head would tell me like oh you’re worthless and things so I’d feel suicidal at school”  Louise: “I hated school, I didn’t really get on with anybody urm, I just kind of was by myself and when I was in a group, I would always try and be like urm, the silly one, who always tries to make everyone laugh, just to try and fit in[ok] but it didn’t work so I would I would get, the voices in my head would tell me like oh you’re worthless and things so I’d feel suicidal at school”  Holly: “yeah …stresses, stressful people, because I like to help people, or listen to people but then I take on their problems and then I’m like wait boundaries [laughs] erm and then, that again is like taking on other people’s commitments or taking on more commitments like physically what you do and the emotion as well”  Holly: “whereas before I would only be honest with mental health professionals and then for example this building, as soon as I left this building, its in there, its in that building, I’m not re-entering that, its not happening”  Belle: it was really scary, it was really scary, urm and he was a dickhead, he really really messed with my head, urm you know I got sent home from work [tearful] it made me feel like shit, and I still worry about it now because its only recent like urm he really affected me, like he really really messed with my head  Belle: so for me, to come in and try and explain to my parents who thought the people that self-harmed were seeking attention seeking, to try and go and explain that to them, they were really confused they didn’t have a clue what was going on, it’s quiet funny looking back at it actually, urm you know, they didn’t have a scooby what was going on do you know what I mean OR as I say like yeah it is attention seeking but not in the way that people say it is, I’m attention seeking because I need help, I need help, I’m screaming out for help like I am not attention seeking because I want people to you know fuss over me or whatever like I am not here for that I was there for, just help me, I need some one to just help me |
|  |  |  |
|  |  | Annie: “well the support network round CAMHS, it is amazing, it is, even though I haven’t had like one counsellor or one psychiatrist the whole way through it’s always…you know, its always up to standards”  Annie: “yeah, but there have been sometimes where I’ve self-harmed but there are sometimes where I have rang them (Crisis team) and probably the reason why I am here today is because I have actually rang them.”  Liz: “yep…and I’m with happy people…personal support (mumbles), I’ve got his family, my family, I’ve got all these people round me and I’ve got some good friends as well, whereas before, I had some really toxic people”  Sarah: “I have been with my partner for five and a half years and we’ve been good throughout the entire time, he’s been very supportive though”  Sarah: “I think we just need to check in on people, you know, if people genuinely sat me down and asked me if I was doing ok, I would have been honest, but nobody asked, you know I am not blaming anyone else or saying that it’s their fault but you know small things like that can really make a difference and if not stop, but at least delay something happening”  Matt: “I mean I’ve been through different therapies and stuff, I genuinely think DBT has helped me through most of my stuff to be fair [ok] ur, like I’ve been speaking to my nurse, they’ve helped me to understand a lot of things, I think I’ve been quite confused and its triggered a lot of emotions while I’ve been trying to think about it, I think it was just being able to kind of go through it all at my own pace, I guess having the skills there for when it did get a bit too much [yeah] I think it did help, urm, yeah, and they kind of helped me”  Matt: “I think that’s what DBT has helped me with ur, especially working with [clinician name] urm I guess being in that 1:1 scenario seeing the difference of how its affected me, talking about it, I think before I just got overly anxious and just really kind of annoyed and ended up kind of zoning out and just getting too annoyed to speak about it [mm] but I think now being able to , I mean especially with [clinician name] I was able to speak about like, especially the bad experiences I’ve had, and its just like being able to accept how angry I got at the time and how it didn’t help”  Louise: “yes it has, like she calls me up every week, like not just to see what drugs I’ve done you know, but just to see if I am ok, which is really nice, urm, its like she actually cares, does that make sense, its like I’m not just another person who’s gone to her for help, this is a girl that I am going to check up on every week to make sure she is ok and that’s how it feels and its really nice to have that”  Louise: “oh yeah, me and mom get on a lot more now, stuff at home is a lot better, um, its still stressful don’t get me wrong but its better than what it used to be… I think having the services I’ve been having and then explaining to my mom what’s going on she kind of understands it a lot more now”  Holly: “definitely, definitely, my support network now is mainly people who either have experiences of mental ill health themselves [mhm] and I can listen to their stories and they listen to mine and we can both kinda go yeah that’s fucked up but I get what vibe you’re on, or I have friends that are, everyone is involved in mental health in some way, like I have friends who are student mental health nurses, I have friends who are, I just, they’re all involved in mental health some way and I think that’s very good because it needs to be spoken about urm so there’s a lot of ability to be honest”  Holly: I think that, with the self-harm suicidality thing, I kept it all in, and I’m a very outward person if I’m with friends or whatever I’m like, how are you doing? What’s going on with you? That kind of stuff and then they’ll ask me about me and I never used to, I used to be like yeah I’m grand thanks carry on, where as in recent years I’ve been more honest and that’s helped a lot  Belle: you know as I say, my mom is my best mate, she’s an amazing mom, like if didn’t have my family I wouldn’t be here, one hundred percent  Belle: I think I can, the one thing I can say is, just when someone is feeling like shit, just talk, that’s all you need, you need to talk out do you know what I mean and I’d like you to make that very obvious because if I hadn’t talk out I wouldn’t be here, I wouldn’t be alive |
| 1. The double-edged sword of alcohol and substances | 2.1 Using alcohol/ substances to escape unwanted emotions  2.2 The adverse impact of alcohol/ substances on mental health  2.3 Others encouraging and normalising use  2.4 The changing and unpredictable use | Annie: “I can a glass of something with my mom and my dad and I’ll be fine, its sort of, it doesn’t numb it, but it just makes it that tad bit easier to cope”  Liz: “probably both, I used to drink and smoke at the same time, it was more the cannabis that made me feel relaxed the alcohol used to make me feel really hyper (laughs)”  Sarah: “I find its (cannabis) kind of a… [long pause] almost like a PRN in the sense of you know, if I find myself feeling a little risky it kind of it just, it lowers my risk, I’m not able to…I can’t think if what I want to say sorry…I think its just kind of the lack of thinking, like I can’t think, I can move but its not, you know its that kind of, I physically am unable to hurt myself if I wanted to, its more effort”  Matt: “its never been something that I’ve gone to when I’m depressed, I’ve got the other self-harming techniques that I grew up with I guess so I guess I dunno drink is just not one of them. Obviously, I had the prosecco when I took my overdose”  Louise: “well I was going through a bit of a rough patch, in my life where I found out that my dad wasn’t my real dad and stuff [right ok], erm and it just kinda, I didn’t really care anymore. So I was just like, I’m gunna start taking drugs and its helps and heals the pain by [mumbles] but in the end it doesn’t, it doesn’t actually help with the pain [right] it just make things worse”  Holly: “I just noticed the effect, like being a student you drink right so, and then you learn about how alcohol affects you then, and if I calms you down, when you want to be calm you be like oh I’ll just have a drink [ok] I realise that’s not healthy but at the time I didn’t care so [ok]”  Belle: as soon as like that happened with him, I just went out drinking and I got drunk every night it was awful and I was just in such a bad way I just wanted to forget about it urm it made me feel worthless, I made me feel like shit |
|  |  | Annie: “I didn’t eat for pretty much that whole two years, I barely ate, I ended up getting, I’ve got an eating disorder now, so its not only that its probably, its ruined my insides, I mean, after I stopped doing solvent abuse, I threw up every day for about 3 months after it, I mean its just….alcohol, if you monitor it, it can help you, I don’t know about weed that much because it sent me paranoid and it messed with my anxiety but solvent abuse, it fucks you up, that’s the best way I can say it.”  Liz: “well I don’t drink quite a bit because it takes me hardly anything to get drunk to be honest (laughs), so I’d just drink like, I dunno… like you know the little bottles of vodka, I’d drink one of them, and then I’d get really hungover, and really regret it, because I made me feel even worse than I felt before (laughs), it didn’t make me feel more depressed, it made me feel happy, but, I just got so hungover, it was horrible”  Sarah: “yeah completely, I was very reliant on it, I found I couldn’t do things unless I’d had a drink, a least a drink, like any time I’d leave the house I would need to have a drink before going out and it just became…I became very angry when I couldn’t drink [ok] and it wasn’t a nice feeling, urm I felt like I wasn’t me, you know, it wasn’t good….  yeah, I think it’s the fact that I’m drinking less that my mental health has improved [ok] urm, I definitely notice, even, even if I go on a night out and I wake up the next morning, my mental health will be…knocked about for a couple of days [right] urm so I know it has a definite effect”  Matt: “the last two times I have smoked with him, I’ve got home and urm become quite psychotic to be fair…overwhelmed with voices, seeing things, urm I just become really restless urm, just cold sweats, yeah just all that and i guess it, especially when I’ve had a smoke I guess it urr, I dunno it doesn’t feel real so it kind of gets me thinking and I mean, its never go so bad its led into self-harm but I’ve thought about it, because of how impulsive it makes me to, I guess its just the overwhelming-ness of the thoughts in your head going at like 100 miles an hour”  Louise: “when I drink erm, I get, obviously I get drunk [yeah] and the normal symptoms and that and then urm, if I continue to drink after that, I go insane, like completely insane, well the once I did it, I went in the shower and I started to self harm with a razor [ok] and my mates were there and they kicked down the door and took my out the shower and then I ran off and tried to jump out of my window to end my life urm, so that was one experience I’ve had, urm, pretty much all similar on drink I just always feel suicidal… always get memories from the past [ok] and urm it just brings everything up and I just, I can’t deal with it so I end up self-harming, I end up trying to take my life or having thoughts of taking my life”  Holly: “I just needed everything to stop because it was a lot, cus I dunno, alcohol calms you down for a bit and then the chaos starts again[ok] and I think I drank myself into chaos so yeah the self-harm was to try and mitigate the alcohol and the alcohol was to mitigate the chaos and it was just a bit of a mess”  Belle: when I drink now, I get anxious really bad after, the day after I mean obviously I went out drinking last night and I am a bit hungover now and obviously that’s why I am getting really upset and I am really emotional and that’s what I am like when I am hungover and that’s the side effects I get when I have alcohol, I get really emotional and I will just cry at everything |
|  |  | Annie: “I didn’t eat for pretty much that whole two years, I barely ate, I ended up getting, I’ve got an eating disorder now, so its not only that its probably, its ruined my insides, I mean, after I stopped doing solvent abuse, I threw up every day for about 3 months after it, I mean its just….alcohol, if you monitor it, it can help you, I don’t know about weed that much because it sent me paranoid and it messed with my anxiety but solvent abuse, it fucks you up, that’s the best way I can say it.”  Liz: “well I don’t drink quite a bit because it takes me hardly anything to get drunk to be honest (laughs), so I’d just drink like, I dunno… like you know the little bottles of vodka, I’d drink one of them, and then I’d get really hungover, and really regret it, because I made me feel even worse than I felt before (laughs), it didn’t make me feel more depressed, it made me feel happy, but, I just got so hungover, it was horrible”  Sarah: “yeah completely, I was very reliant on it, I found I couldn’t do things unless I’d had a drink, a least a drink, like any time I’d leave the house I would need to have a drink before going out and it just became…I became very angry when I couldn’t drink [ok] and it wasn’t a nice feeling, urm I felt like I wasn’t me, you know, it wasn’t good….  yeah, I think it’s the fact that I’m drinking less that my mental health has improved [ok] urm, I definitely notice, even, even if I go on a night out and I wake up the next morning, my mental health will be…knocked about for a couple of days [right] urm so I know it has a definite effect”  Matt: “the last two times I have smoked with him, I’ve got home and urm become quite psychotic to be fair…overwhelmed with voices, seeing things, urm I just become really restless urm, just cold sweats, yeah just all that and i guess it, especially when I’ve had a smoke I guess it urr, I dunno it doesn’t feel real so it kind of gets me thinking and I mean, its never go so bad its led into self-harm but I’ve thought about it, because of how impulsive it makes me to, I guess its just the overwhelming-ness of the thoughts in your head going at like 100 miles an hour”  Louise: “when I drink erm, I get, obviously I get drunk [yeah] and the normal symptoms and that and then urm, if I continue to drink after that, I go insane, like completely insane, well the once I did it, I went in the shower and I started to self harm with a razor [ok] and my mates were there and they kicked down the door and took my out the shower and then I ran off and tried to jump out of my window to end my life urm, so that was one experience I’ve had, urm, pretty much all similar on drink I just always feel suicidal… always get memories from the past [ok] and urm it just brings everything up and I just, I can’t deal with it so I end up self-harming, I end up trying to take my life or having thoughts of taking my life”  Holly: “I just needed everything to stop because it was a lot, cus I dunno, alcohol calms you down for a bit and then the chaos starts again[ok] and I think I drank myself into chaos so yeah the self-harm was to try and mitigate the alcohol and the alcohol was to mitigate the chaos and it was just a bit of a mess”  Belle: when I drink now, I get anxious really bad after, the day after I mean obviously I went out drinking last night and I am a bit hungover now and obviously that’s why I am getting really upset and I am really emotional and that’s what I am like when I am hungover and that’s the side effects I get when I have alcohol, I get really emotional and I will just cry at everything |
|  |  | Annie: “now I have a few drinks now, I go to parties, I don’t get obliterated, cuz I don’t know whether I’m going to be a happy drunk or an angry drunk, I don’t know, but I don’t push myself to that limit but alcohol, its fine with me, I can a glass or something with my mom and my dad and I’ll be fine, its sort of, it doesn’t numb it, but it just makes it that tad bit easier to cope”  Annie: “I don’t get obliterated, cuz I don’t know whether I’m going to be a happy drunk or an angry drunk, I don’t know, but I don’t push myself to that limit but alcohol”  Sarah: “yeah sometimes, its not as dark, but I try to like, now that I’m aware of my warning signs you know if, if I’ve had a drink I kind of need to check in with myself, am I going to be able to keep drinking, do I need stop, like try to kind of stop it before it gets too low”  Sarah: “it just kinda got me out my head, sometimes, you know I’d get really giggly and I’d watch comedy shows, like have a laugh, [quietly] the other times it would be really bad. There was no, kind of in between, by the end of the night I was either…high with joy or completely just depressed, there was nothing in between.”  Matt: “yeah yeah it was and that’s why I’ve kind of had to stop kind of going to see my cousin because its just about being tempted, I, I guess, its not like I’ve got a drug problem, I think cus growing up it made me quite chilled out and it was quite a regular thing for me and my cousin to just chill really”  Matt: “its never been something that I’ve gone to when I’m depressed, I’ve got the other self-harming techniques that I grew up with I guess so I guess I dunno drink is just not one of them. Obviously, I had the prosecco when I took my overdose”  Louise: “Drugs I, I am trying with the drugs, it’s the drink that I’ve kind of cut down [yeah] and realised its just because the cannabis is so, has such a big impact on me, it keeps me so calm [right] its hard to stop something that helps you, whereas I know from other drugs I cant take them because it will either react with my medication or it will just send me to suicide [yeah] so I tend to just know my limits and keep myself safe in a way”  Louise: “if I’m having a bad day, or it heightens my voices cus normally weed doesn’t heighten my voices, it takes it away, it reduces it [mm] but sometimes it can heighten it, yeah so, it’s really hard to explain [no, you’re doing a good job] its just one of them things really where it helps but it doesn’t help sometimes”  Holly: “very conscious of that, very conscious of anything that goes in my body anymore”  Belle: so the drugs came first, it was the weed that came in first really, you know I didn’t touch any class As or anything until I was about 18, 19, urm it was, it was weed, then it was alcohol and then it was class As urm, obviously weed is a gate, gate drug anyway, gateway drug which is really awful |
| 1. The straw that broke the camel’s back | 3.1 A gradual build up  3.2 Being determined to harm the self | Annie: “people push you to do it, or things push you to do it, do you know what I mean, there’s only so far you can stretch an elastic band before it snaps”  Annie: “you can either stop yourself from it or you do it and if you are doing it, it just proves how much you are genuinely hurting because I mean how much pain does someone have to be in to physically hurt themselves?”  Sarah: “Urrm…the first one was when I was, I’m going to saying 15, it’s either 14 or 15, urrmm…and it was just like a mild overdose [ok], urmm…that was the first one, there’s been a couple of, I would say minor urm, instances of, you know, self-harming attempts or minor overdoses, nothing serious and the last time was urm, when I was 21 [ok], urm and…it was quite severe urm, there was self-harm and an overdose…”  Sarah: “well I was being…quite severely bullied in school, so…again that was a kind of build up, I had urm long broke up with my girlfriend at the time, and it just seemed to be that that was the trigger for everything else [yeah] on top of it, I guess at that point”  Matt: “I mean I spent probably about three weeks on my dad’s sofa [right], urr before any self-harm, I wasn’t eating, wasn’t drinking, I, I think I went down to about six stone [gosh]…yeah so I mean it, it, it’s weird to think back now, but it, I lost a lot of weight”  Matt: “I mean there was a few things that happened at the time, urr, I had a girlfriend, I was doing, well I was over working myself, I was doing about 80 hours at work [oh gosh] urr and I was going to college as well (laughs), so yeah, urr, it was just everything coming to an end really, cus I was working so much, I lost my college placement [right] and then I got over tired and I got urr, well I had to had in my notice cus of things that were happening, and my relationship ended so…urr it was just everything kind of coming crashing down”  Louise: “erm, I’ve self-harmed for quite a while now, it’s on and off [yeah] urm, I don’t do it every day, I do it every what I probably do it twice every three months [ok] now but that’s because of the help I’ve been getting [yeah] so, I’m quite proud of that”  Holly: basically when I self-harmed I think I hallucinated, I’m not sure at this point [that’s ok] but I think I did because all I can see was purple, and obviously blood is red and there was a lot of blood not to be graphic, but urm I was seeing purple and I thought it was electricity so I thought there as electricity coming out of me so yeah its mental to say it// I hadn’t slept in 6 days by that point [ok] so I think that was a major factor in something urm and like when I drank before I thought oh alcohol helps you sleep so I’ll just drink alcohol to sleep and then it didn’t so my energy must have been going for it at that point but it was really interesting [mumbles]  Belle: I was, I was self-harming constantly, I was constantly self-harming and urm I mean it sounds really stupid but you know the TCP oil, now we can’t have that in house now ever, because when I was younger that’s what we would clean my self-harming with because obviously it’s an antiseptic and I used to use dirty razors |
|  |  | Annie: “I tried to overdose using tablets, and there was one where I ended up inhaling solvents to try and end my life because of, I saw on the deodorant can it said solvent abuse can kill instantly”  Louise: I’ve tried strangling, I’ve tried urm, hanging myself, jumping, I’ve tried a few things to be fair and whether that’s because of drugs I’ve taken in the past and how its affected my body, I’m not sure  Belle: then there was other attempts like with the bleach and I was going to take an overdose again, again I was planning to hang myself or I used to plan a lot, falling down the stairs, trying to, which I know sounds really stupid, but you know, you’d really injury yourself more than dying falling down the stairs but, I used to think, if I get the right trip, I can die and I remember I used to google how to break your hand and stuff like that like to try and hurt myself, urm and I used to jump off the bed to try and hurt myself, to try and break an arm or really really harm myself, where I could be in a cast for like 6 weeks you know, I was trying to do stuff like, and urm you know the thought of jumping of a bridge or anything, I’d be petrified because I am petrified of heights so I would never do that and I never thought about doing it either, I think mainly it was taking something that I thought would be the best way |
| 1. Reflecting on the on-going process of recovery | 4.1 Increasing understanding of the self and experiences  4.2 Using alternative coping strategies  4.3 Taking responsibility for recovery  4.4 The emotional difficulty of reflecting | Sarah: “I try to kind of…do a lot of self-care, but not in a sense of bath bombs or face masks but in a sense of like, ok checking in with myself and seeing ok, if I am in a bad mood, have a showered today? Did I wash my hair, have a brushed my teeth? Have I eaten anything, drank anything? I used little things like that to kind of check in…you know, you might be angry and realise you haven’t had breakfast, you have breakfast, its all sorted. I think things like that are really kind of overlooked, urm so I try my best to kind of keep on top of the body maintenance, mind maintenance, you know that kind of things”  Matt: “it’s taken up to now to kind of realise everything that’s gone on… yeah, urr, I spent the last couple of years, urr doing therapy, speaking to professionals, I mean it hasn’t always gone the best because I guess its just frustrating sometimes when you’re trying to get better yourself”  Matt: I think that’s what DBT has helped me with ur, especially working with [clinician name] urm I guess being in that 1:1 scenario seeing the difference of how its affected me, talking about it, I think before I just got overly anxious and just really kind of annoyed and ended up kind of zoning out and just getting too annoyed to speak about it [mm] but I think now being able to , I mean especially with [clinician name] I was able to speak about like, especially the bad experiences I’ve had, and its just like being able to accept how angry I got at the time and how it didn’t help  Louise: “yes they have, that’s another reason why I’ve kind of stopped alcohol because they’ve you now told me about it and I’ve just realised that alcohol doesn’t help at all”  Holly: this time last year and this time last year I was in a very like dark depression, like very bad, I would say the worst I’ve ever been [ok] but I dealt with it in a very different way whereas before I would just give all of my power to doctors, nurses and mental health professionals generally whereas this time I was like, I am surrounded by people I love, I’m safe, as safe as possible and I looked at it very differently  Holly: I think it is very representative, I think it is very representative of a lot of people, a lot of young women, to get political urm who have a diagnosis of borderline personality disorder, personally I disagree when the young person is going through puberty, the emerging bit exists for a reason but I think that has a big factor because I was diagnosed with BPD, EUPD whatever you want to call it and then, when I went home and did my research I was like, what the fuck are you on about? This isn’t me, what, I don’t know what you’re on about urm, and then when I got the bipolar diagnosis initially, I was like oh this answers everything oh my god  Belle: I know it sounds really bad but if I didn’t go through what I went through, I wouldn’t’ be the person I am today, you know I have got a full time job, I’m going out, working, I do 40 plus hours a week, I’m not taking tablets anymore, its around about a year now that I’ve stopped taking tablets  Belle: when he explained it to me and what you know what it meant, it was mad because, it just connected in my head, I was like shit, that is, that it what it is you know, going through those times, you know up and down all the time it was so difficult |
|  |  | Annie: “I started smoking cigarettes and vape, urm I started smoking cigarettes when I was like 11, urm and that was like a coping mechanism and then I ended up stopping when I went into year 10, didn’t work and then I got a vape and I use my vape now, but apart from that I’ve got four cats, that I just try and surround myself with them”  Liz: “R: so are there particular skills that are really helpful do you think?  Liz: Interpersonal skills ‘cus, I can be like…I get anxious in like when I talk to people sometimes, mostly not family and friends but it’s like confusing stuff (laughs), like when I’m phoning up at work, phoning somebody to sort something out, it stresses me out, so I just use the interpersonal skills then…”  Sarah: “I don’t know, I just stopped drinking, urm, I go to the gym, I try to eat well…it’s the little things that everyone rolls their eyes at that have actually all made a little impact [yeah] and I just seem to be kinda taking better care of myself and standing up for myself urm, so that’s kind of given me that boost, do you know what I mean?”  Matt: “having that 1:1 and having that idea on mindfulness and how it work I was able to use it in kind of everyday activities that I were doing so using it at the gym, being mindful at the gym [yeah] ur, ur, even when I was smoking, I mean I quit recently but just having a, it sounds stupid but having a mindful cigarette [mm] not just smoke it because it’s a habit but sit there and thinking err just thinking about things I guess and putting things in perspective and not, err, letting it overwhelm me all the time”  Louise: “I go to DBT at the moment…that’s going alright, I’ve got two more sessions left [mhm], they’ve taught me skills and how to cope and stuff like that so urm, that, I’ve also got that, which is why I’ve only been doing cannabis every three weeks, because I’ve been using skills”  Holly: “I go to the gym a lot, and I go for walks and I talk to people and I don’t delve into impulses like before”  Belle: I do smoke, so I’ll go for a fag and I’ll calm myself down…I’ll sit and watch youtube videos, which I know sounds really stupid but I will sit and watch make up videos or something on youtube and that’ll [clicks fingers] |
|  |  | Liz: “I needed to sort myself out, I don’t think there’s anything that could have been done I just needed to sort myself out”  Sarah: “I think it was, it was on me, I was very dismissive of any kind of issues I had, I didn’t wanna…burden anyone [ok], urm and I just let it build up, urm, so I think, honestly it was kind of on me”  Sarah: “anyone who would listen, I didn’t wanna put it on anyone, I didn’t wanna…bother anyone, urm, it just seemed like you know, that’s your shit you’re just going to have to deal with it, don’t get anyone else involved, in my head that made sense at the time”  Louise: “that’s something I’ve got to deal with considering I’m the one taking drugs, i’ve got to be prepared for that”  Holly: “because I remember a nurse in hospital about 3 and a half years ago, she said to me you need to take responsibility and I was like what the fuck are you on about? You’ve got responsibility over me right now like on a section and all that kind of stuff, you’ve got responsibility over me I haven’t gotta do anything and it wasn’t until my last depressive episode I was like oh that’s what she means, I have to do stuff too, I’m not just a passive person that takes medication and then leaves hospital”  Belle: I think because I am a positive person which helps, I think you’ve really got to be in the right mindset especially with mental health like, if you’re not in the right mindset it’s not going to work, its really not, you need to you know, sit yourself, what I do is I just sit myself down |
|  |  | Annie: “you start talking, you’re opening old wounds, you’re gunna feel like shit but I mean I’ve been coming to CAMHS since I was in year 7, I’ve sort of gotten used to the whole things of talking about it and that’s what people need to realise, you can’t just hold it in, you’ve gotta talk”  Liz: “R: and were there many other ways that you tried to end your life apart from overdoses? Liz:…yeah…R: ok Liz: quite a few, but I don’t want to talk about it”  Sarah: “yeah urm…yeah, it’s just not, it not a nice thing to be thinking about”  Matt: I think that’s what DBT has helped me with ur, especially working with [clinician name] urm I guess being in that 1:1 scenario seeing the difference of how its affected me, talking about it, I think before I just got overly anxious and just really kind of annoyed and ended up kind of zoning out and just getting too annoyed to speak about it [mm] but I think now being able to , I mean especially with [clinician name] I was able to speak about like, especially the bad experiences I’ve had, and its just like being able to accept how angry I got at the time and how it didn’t help  Louise: “yeah, I just can’t stand it, I don’t like telling anybody at all…  yeah just I don’t want people to think I am not coping well”  Belle: you know as a kid I was such, I’m a bubbly person, but I lost myself for so long in that time, urm [tearful] sorry…looking back at that time, it is, it’s really, it’s difficult but I’m glad I went through it”  Annie: “…and looking back on it, it just, it makes me sad but it also makes me feel sort of angry that I was, you know, caning eight of them cans a day, one after the other, not stopping until its all gone, every day for about two years and it didn’t kill me”  Liz: “urrm, its happened multiple times, in different ways, it was like a really stressful experience and something like I really regret now”  Matt: it amazes me to be fair, urr…I thought saw so negative to lead me to doing something like that, [mmm], urr…and I can’t quite understand what led to me actually doing it |
