# Supplementary material for: A qualitative exploration of young people’s experiences of attempted suicide in the context of alcohol and substance use
Source: PLoS One. 2021 Aug 31;16(8):e0256915. doi: 10.1371/journal.pone.0256915 (PMC8407575; doi:10.1371/journal.pone.0256915)
Supplement: Attachment — Submitted filename: Response to reviewers.docx [file pone.0256915.s007.docx]

Dear reviewers,

Thank you for taking the time to read this paper and for your thoughtful comments and suggestions. Please see the table below which outlines your comments and the responses.

| Change requested | Response | Line number |
| --- | --- | --- |
| Please ensure that your manuscript meets PLOS ONE's style requirements, including those for file naming. The PLOS ONE style templates can be found at  <https://journals.plos.org/plosone/s/file?id=wjVg/PLOSOne_formatting_sample_main_body.pdf> and  <https://journals.plos.org/plosone/s/file?id=ba62/PLOSOne_formatting_sample_title_authors_affiliations.pdf> | Style requirements and formatting changed in line with guidance provided. |  |
| In your Data Availability statement, you have not specified where the minimal data set underlying the results described in your manuscript can be found. PLOS defines a study's minimal data set as the underlying data used to reach the conclusions drawn in the manuscript and any additional data required to replicate the reported study findings in their entirety. All PLOS journals require that the minimal data set be made fully available. For more information about our data policy, please see http://journals.plos.org/plosone/s/data-availability.    Upon re-submitting your revised manuscript, please upload your study’s minimal underlying data set as either Supporting Information files or to a stable, public repository and include the relevant URLs, DOIs, or accession numbers within your revised cover letter. For a list of acceptable repositories, please see http://journals.plos.org/plosone/s/data-availability#loc-recommended-repositories. Any potentially identifying patient information must be fully anonymized.    Important: If there are ethical or legal restrictions to sharing your data publicly, please explain these restrictions in detail. Please see our guidelines for more information on what we consider unacceptable restrictions to publicly sharing data: <http://journals.plos.org/plosone/s/data-availability#loc-unacceptable-data-access-restrictions> . Note that it is not acceptable for the authors to be the sole named individuals responsible for ensuring data access.    We will update your Data Availability statement to reflect the information you provide in your cover letter. | Minimal dataset, which includes participants’ quotes, has been uploaded as Supplementary information. | See appendix 6 and line 187 |
| Please amend either the title on the online submission form (via Edit Submission) or the title in the manuscript so that they are identical. | Manuscript title amended. | Line 3 |
| Please include captions for your Supporting Information files at the end of your manuscript, and update any in-text citations to match accordingly. Please see our Supporting Information guidelines for more information: http://journals.plos.org/plosone/s/supporting-information | These have been added to the end of the manuscript as requested. | See throughout text and captions at the end of manuscript |
| ABTRACT |  |  |
| It is somewhat difficult to follow the themes placed in quotations. I would suggest that the authors either find new ways to describe and/or organize their key themes | We have now revised the abstract to improve readability of the key themes. | From line 22 |
| This manuscript would be greatly improved if the authors detailed the percentage of participants who endorsed a given theme | Percentages now included in results section. | Table 2, line 189 |
| The abstract highlights barriers. Were facilitators also explored? | In the abstract, we report inter and intrapersonal factors which impact upon young people’s suicidal experiences including suicidal ideation and attempts. Barriers and facilitators were not explored in this study. |  |
| INTRODUCTION |  |  |
| The authors highlight risk factors of suicide. Are these limited to completed suicide or also relevant for ideation? | The risk factors reported refer to completed suicide. | Line 53 |
| Can a sentence or two also highlight protective factors? | Please see line 56 | Line 56-58 |
| It is not clear how the authors differentiate ‘alcohol’ and ‘substance use’ | For the purpose of this study, the term ‘substance use’ is used to describe any illicit drug use, not including alcohol. | Line 87-88 |
| Please add appropriate references for statistics: e.g., “Young people (over 18) who engage in binge drinking have been found to be 2.6 times more likely to attempt suicide” | We have removed this sentence and have now clarified that “Esang & Ahmed (2018) [14]2reported that drinking alcohol at a young age, alongside binge drinking leads to increased suicide ideation in adulthood. They explain that those with alcohol dependence or substance use are more than 10 times more likely than the general population to die by suicide.” | Line 66-70 |
| METHODS |  |  |
| The authors list that having a ‘self-defined problematic use of alcohol (and substances’ was required as inclusion criteria; however, it is known that adolescents may not recognize that they have issues with substances, especially alcohol. Can the authors justify why this criteria was required? | Please see line 124 for further explanation of this decision. | Line 124-127 |
| The authors exclude those who have made a suicide attempt in the past 6 months. Why was this the case when the authors previously argue that it is important to examine those with alcohol issues to inform suicide interventions? | In line with advice received by the Research Ethics Committee, those who have made an attempt in the past 6 months were excluded as a way of minimising distress involved with taking part and managing individual risks so close to the event. | Line 130-131 |
| Can the authors justify why they used a sample size of 7 and not less or more? Was saturation of specific themes considered for the listed sample size? | The literature suggests that 4-10 participants is a sufficient number for an IPA study (Smith et al, 2009). The sample size of 7 is in line with the above literature and allowed for a thorough and in-depth exploration of young people’s experiences. | Line 152 |
| Interviews being conducted by the local mental health team may introduce bias. Independent and trained research personnel are typically the gold standard for qualitative research. A rationale for why this interviewee was selected for the current study should be detailed and potentially recognized as a limitation in the Discussion section. | Please see line 133 and 147, all interviews were conducted by lead researcher (RG), as is the gold standard. These interviews took place at the local teams were participants were familiar with the setting. | Line 133 and 147 |
| Who conducted the debrief session that followed the qualitative interview? | None of the participants requested a debrief. This was being offered by a psychologist independent of the research study. | Line 143-144 |
| It is not clear who conducted the analysis and whether inter-rater reliability was reached prior to completing all analyses | Lead researcher RG analysed the results which were then discussed with qualitative peer group and co-authors to ensure reflexivity and credibility of findings. | Line 147 and line 163-165 |
| RESULTS |  |  |
| Table 2 is informative however it is not clear what the third column (“Participants that contributed”). These results could be bolstered by highlighting the frequency and percentage of participants who endorsed a given theme | Percentages added to table 2 | See line 189 |
| The theme “Keeping safe from others’ does not seem to appropriately reflect the longer text. It sounds like all participants felt misunderstood by people around them | We thank the reviewer for this comment. The term keeping safe from others was chosen as it was felt to encompass both the consequences and stigma related to not being understood by others but also the physical and emotional harm that some participants have experienced at the hands of others. |  |
| It may worthwhile to organize the themes accordingly to barriers and facilitators. This organization is particularly informative to interventionists. | We thank the reviewer for this suggestion. However, identifying barriers and facilitators was not the aim of the study. Within an IPA framework the results are analysed to understand individual and shared experiences and are therefore written in a way to best describe these. Recommendations that might speak to interventions is discussed within the clinical implication sections of the discussion. |  |
| It is not clear whether the data was analyzed for stratified groups e.g., younger vs. older youth; male vs. female | This is unfortunately not possible within an IPA framework and a relatively small sample size. |  |
| DISCUSSION |  |  |
| Additional limitations including the small sample size should be noted. It is not clear whether these results are generalizable to contexts outside of the UK or to other, non-white groups | IPA methods are not focused on generalisability in the same way as quantitative research. IPA findings aim to enhance understanding, increase insight and make a step in the contribution to existing theories and the generation of new hypotheses to be further tested in future studies. As such, the study findings provide new insights from participants’ perspectives to be further investigated. | Line 648-651 |

Yours sincerely,

Rebecca Guest
